# Development of Innovative Gluten-Free and Egg-Free Pasta from Acorn Flour and Carob–Xanthan Hydrogel

**DOI:** 10.3390/gels12070610

**Published:** 2026-07-08

**Authors:** Francesca Vurro, Alexandra-Mihaela Ailoaiei, Giacomo Squeo, Francesco Caponio, Antonella Pasqualone

**Affiliations:** Department of Soil, Plant and Food Science (DISSPA), University of Bari ‘Aldo Moro’, Via Amendola, 165/a, 70126 Bari, Italy; a.ailoaiei@phd.uniba.it (A.-M.A.); giacomo.squeo@uniba.it (G.S.); francesco.caponio@uniba.it (F.C.)

**Keywords:** rheological properties, texture, bioactive compounds, cooking behavior

## Abstract

The increasing demand for sustainable, plant-based, gluten- and egg-free options is stimulating innovation in pasta products. The present work was aimed at investigating the effect of acorn flour (AF) at 50% (A50) and 100% (A100) in gluten-free and egg-free clean label *tagliatelle*, compared to a rice flour-based version (CTRL). A hydrogel consisting of carob seed flour and xanthan gum was used to reproduce the viscoelastic properties of gluten. Samples were analyzed for their rheological, physicochemical, nutritional, and sensory properties. The use of AF elevated the elastic modulus (G′), phenolic content, antioxidant activity, lipid content, and fiber content. The sample A100 was a “source of fiber”, according to EC Reg. 1924/06. In terms of cooking behavior, the incorporation of AF induced an increase in the water absorption index (WAI), the swelling index (SW), and a higher cooking loss. The addition of AF also resulted in a greater firmness at the cutting test and a brown color. The acorn *tagliatelle* had a fruity odor and flavor note. Based on these findings, AF could be a valid option in novel functional food prototypes, and also in gluten-free, egg-free and vegan versions.

## 1. Introduction

Pasta constitutes a cornerstone of the Mediterranean diet and Italian gastronomic traditions [1].

The provenance of this food remains a topic of considerable debate. One school of thought attributes its introduction by Arabs in Sicily, while another believes that it was Marco Polo who first imported Asian noodles to Europe. Nonetheless, the prevailing hypothesis propounds that the introduction of this food was initiated by the ancient Greeks and Romans, who prepared it as strips made from flour and water, denominated “*lagane*”, and consumed it boiled in combination with legumes and vegetables, as documented by the poet Horace [2,3].

From a technological perspective, pasta is classified as fresh and dried, based on formulation, processing and stability. Fresh pasta is characterized by high moisture content (≥24%) and high water activity (a_w_ 0.92–0.97), which results in limited shelf-life and necessitates the application of preservation strategies such as pasteurization followed by refrigerated storage [4]. The process of producing fresh pasta generally includes the following steps: first, a mixture of wheat flour, water and, frequently eggs, is prepared; then the mixture is extruded or laminated, followed by cutting or shaping into long pasta (e.g., *tagliatelle*, *tagliolini*), short pasta (e.g., *maltagliati*), and flat sheets for lasagna or for the wrapping of fillings, including cheese, vegetables, and meat [1,3].

Conversely, dried pasta is commonly produced exclusively from wheat flour, predominantly durum wheat semolina, and water. The dough is shaped mainly by extrusion and dried under controlled conditions designed to decrease the level of moisture to ≤12.5%, ensuring microbiological stability and an extended shelf-life at room temperature [3,4].

The pasta portfolio is already extensive in shapes, names, and adaptability to different sauces. However, a multitude of factors are contributing to its continued growth, including health concerns (e.g., coeliac disease and egg allergies), ethical considerations (e.g., vegan diets), and emerging trends promoted by social media, such as clean eating, ketogenic (a low-carbohydrate, high-fat dietary pattern) and plant-based diets, as well as other viral food trends (e.g., sport-oriented diets, high-protein products, and social media-inspired recipes) [5,6].

The exclusion of gluten and eggs from pasta raises several technological issues, such as loss of structure and elasticity, pale appearance, and limited integrity during cooking [7]. In order to overcome these challenges, the use of hydrogels, which consists of a three-dimensional network of hydrophilic polymers, has been demonstrated to be useful in reproducing the gluten matrix [8]. Carob (*Ceratonia siliqua* L.) pods contain high concentrations of polysaccharides, offering technological functional benefits in food applications. Furthermore, this Mediterranean legume plant exhibits a high degree of resilience to extreme environmental conditions, requiring minimal water and nutrient inputs, thereby adapting to conditions that are typically extreme for most crops [9]. Similarly, xanthan gum, a high-molecular-weight exopolysaccharide produced via *Xanthomonas campestris* fermentation, aligns with biotechnological development and has versatile food applications [10,11]. In this regard, the combined use of carob seed flour and xanthan gum is a promising solution, offering a dual benefit in addressing both technological functionality and ethical sustainability [8,12]. This also aligns with the clean label concept, which is characterized by a concise and easily comprehensible list of ingredients.

Hydrocolloids, which act as binders, are added to gluten-free flour, usually rice or corn. Recently, acorn flour, which is also gluten-free, has been considered for its functional properties [13]. Furthermore, acorns are an abundant but not yet adequately exploited resource, the use of which could enhance the value of the forest areas from which they originate, where agricultural activities are not possible.

Previous studies have successfully tested acorn flour in the development of pasta and noodles, but without considering specific categories of consumers, such as celiacs, the egg-allergic or vegans [14,15,16]. In this framework, the present research aimed to (i) develop and characterize egg-free and gluten-free clean label *tagliatelle* formulated from rice flour (RF) and acorn flour (AF), using a carob seed flour–xanthan gum hydrogel to improve dough rheological properties and technological performance, and (ii) promote AF as a resilient, sustainable, and nutrient-rich ingredient for “free-from” pasta.

## 2. Results and Discussion

### 2.1. Rheology of Dough

The rheology of dough is of critical importance for the quality of pasta. This is particularly true for gluten-free recipes, which are already characterized by significant textural problems [17]. Hydrogels can contribute to gluten-like network formation by providing a viscoelastic structure [18]. In this study, carob–xanthan hydrogel was used.

**Figure 1:**
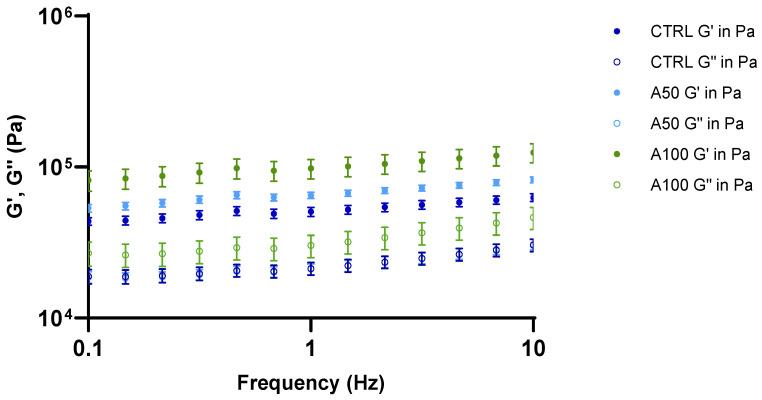
Mean storage modulus (G′, Pa) and loss modulus (G″, Pa) of doughs. CTRL = *tagliatelle* 100% rice flour; A50 = *tagliatelle* 50% rice flour–50% acorn flour; A100 = *tagliatelle* 100% acorn flour. Data are presented as means ± SD of three replicates.

The effect of AF at 50% (A50) and 100% (A100) on dough rheology was compared to that of a rice flour-based dough prepared as the control (CTRL). For all doughs analyzed, G′ was higher than G″ across the range of frequencies tested, and both moduli increased with increasing frequency (Figure 1). Compared to the CTRL dough, the incorporation of AF increased the elasticity, as evidenced by the higher G′ value. The value of tan δ calculated at 1 Hz was 0.42 ± 0.01 in CTRL, which was higher than the 0.32 ± 0.01 and 0.31 ± 0.01 values observed in A50 and A100, respectively. These results confirmed the observations related to the storage modulus G′ discussed above. The RF-based dough presented a higher viscous contribution compared to the other samples, while the samples with AF demonstrated higher elasticity.

This phenomenon has been ascribed to multiple factors related to the chemical properties of AF. This ingredient is characterized by a high percentage of carbohydrates, represented primarily by starch and dietary fiber [19]. Starch, the main carbohydrate fraction, can form networks with other dough constituents, contributing to a solid-like structure [20,21]. Fiber can interact in this network, resulting in stronger elastic behavior [20]. In fact, previous studies have shown that AF in dough causes a significant increase in the elastic modulus, acting as a strengthening agent [22].

In addition to polysaccharide interactions, other AF components may also be involved. Phenolic compounds of acorns, particularly tannins, can interact within the dough matrix, leading to non-covalent bonds with amylose [23]. Furthermore, tannins are well-known for their high binding affinity toward different protein structures, which can induce protein–tannin complex formation [24]. These interactions can further reinforce the polymer network, thereby increasing elasticity and stability [23,25].

To quantitatively support these observations, correlations between the elastic modulus G′ and selected chemical components, discussed in the following paragraphs, were calculated. A strong positive correlation was observed between G′ and fiber (r = 0.96, *p* < 0.001). Similarly, G′ showed a strong correlation with total phenolic compounds (r = 0.95, *p* < 0.001) and tannins in raw pasta (r = 0.90, *p* < 0.001), suggesting a close relationship between these components and the elastic behavior of the dough.

**Figure 2:**
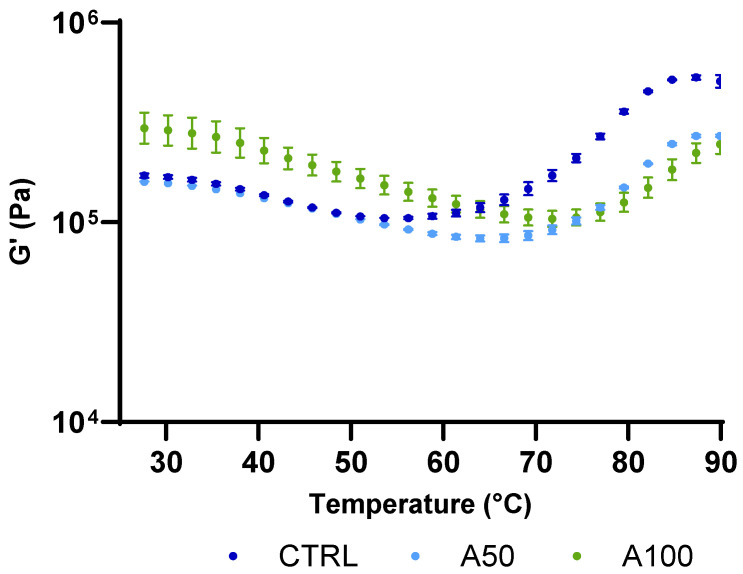
Mean storage modulus (G′, Pa) of doughs as a function of temperature (T, °C). CTRL = *tagliatelle* 100% rice flour; A50 = *tagliatelle* 50% rice flour–50% acorn flour; A100 = *tagliatelle* 100% acorn flour. Data are presented as means ± SD of three replicates.

The rheological behavior of doughs during the heating process was evaluated at a frequency of 1 Hz, considering a temperature ramp from 25 to 90 °C (Figure 2), which is useful for comprehending the structural changes during the cooking process of pasta.

During the initial heating stage (<65 °C), the dough decreased in elastic modulus G′. In these conditions, the starch granules retain water and swell, causing an increase in viscosity, and a low peak of G′. As the temperature increased (65–90 °C), a high G′ peak was reached.

The samples showed different behavior during thermal treatment. This phenomenon was probably correlated with the chemical characteristics of starch, specifically the ratio between amylose and amylopectin. Additionally, it could be influenced by gelatinization temperatures, as well as the interactions of starch with lipids, fiber, and proteins [26,27]. Previous studies have suggested that acorn starch gelatinizes around 60 °C [22], while rice flour gelatinizes at around 70–75 °C [28]. In the first stage of treatment, the two samples with RF presented a similar trend, diverging from the 100% AF sample. Then, the two samples with AF showed a more similar rheological response, significantly differentiating from the CTRL.

### 2.2. Nutritional and Chemical Properties

As detailed in Table 1, the water activity of all samples showed similar values, lower than or equal to 0.97, the maximum value accepted for fresh pasta [4].

All of the experimental samples met the requirements for fresh pasta, with a moisture content of at least 24% [4]. The slight reduction in moisture observed in *tagliatelle* with AF could be connected to the different capacity of flours to retain water.

The addition of AF markedly influenced the nutritional composition of pasta. The most pronounced change was the significant increase in lipid, ash, and fiber concentrations. This enrichment was a direct consequence of the chemical composition of AF, which is known to contain high levels of these nutrients [29]. This trend is in line with the findings of Konya et al. [15] in acorn-enriched gluten-free noodles.

The addition of AF enriched the pasta in fiber, and sample A100 was classified as a “source of fiber” based on Regulation (EC) No 1924/2006 [30], because it was higher than 3 g/100 g of pasta. This represented a relevant nutritional improvement, given the well-documented role of fiber in promoting gut health and metabolic regulation, particularly in gluten-free products, which are often characterized by low fiber levels.

A significant reduction in protein content was caused by the inclusion of AF, in line with several studies on acorn-based products [22,31]. However, this decrease does not necessarily represent a nutritional limitation, as pasta is often seasoned with protein-rich meat or vegan sauces. From a product development perspective, protein levels in acorn-enriched pasta could be optimized through strategic formulation. This can be achieved by partially replacing flour with protein-rich ingredients, such as legume flours or protein concentrates [32].

The energetic value of *tagliatelle* was boosted by the inclusion of acorns, due to the higher lipid content of AF than RF. However, the results were overall in line with the mean value of commercial products, which are generally below 300 kcal/100 g depending on the recipes [33].

**Figure 3:**
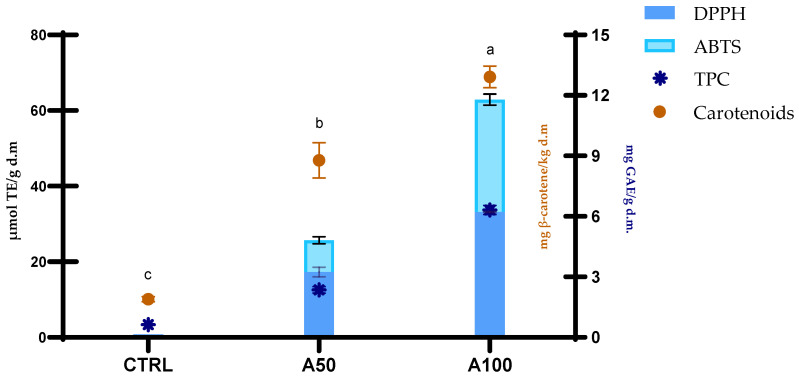
Bioactive compounds of *tagliatelle* pasta samples. CTRL = *tagliatelle* 100% rice flour; A50 = *tagliatelle* 50% rice flour–50% acorn flour; A100 = *tagliatelle* 100% acorn flour. d.m. = dry matter; TPC = Total Phenolic Compounds; ABTS = 2,2′-azino-bis-3-ethyl benzthiazoline-6-sulphonic acid; DPPH = 2,2-diphenyl-1-picrylhydrazyl; GAE = gallic acid equivalent; TE = Trolox equivalent. Carotenoids are expressed as mg β-carotene/kg d.m.; TPC as mg GAE/g d.m. Antioxidant activity as μmol TE/g d.m. Data are presented as means ± SD of three replicates. Different letters indicate significant differences at *p* ≤ 0.05.

The incorporation of AF enhanced the antioxidant activity of *tagliatelle*, a phenomenon primarily ascribed to the high concentrations of phenolic compounds and carotenoids (Figure 3). These compounds are well-known for their strong capacity to mitigate oxidative stress, a process that can lead to cellular damage and contribute to the development of chronic diseases [13,34].

The results indicate that the antioxidant activity was almost negligible in formulation CTRL, as is typically observed in products based on refined flours [35]. Conversely, incorporating AF caused a substantial increase in antioxidant levels in direct proportion to the percentage used. This dose-dependent increase highlights the potential of AF as a valuable means of developing functional foods with enhanced health-promoting properties [13,29,34].

The tannin content detected in *tagliatelle* is reported in Table 2. In the CTRL rice-based formulation, tannins were only detected in the raw sample, in a very limited quantity, possibly due to the tannins naturally present in the carob seed flour used in the hydrogel [9]. However, their presence was no longer identified after cooking. The measured levels in acorn-based *tagliatelle* were in line with those generally reported for acorn flours from different *Quercus* species [32,36]. The commercial acorn flour used in this study was probably obtained from relatively sweet acorn varieties or subjected to a preliminary industrial debittering process [29]. Indeed, the traditional approach to processing acorns involves a series of procedures aimed at reducing their astringent taste and potential adverse digestive effects. These include prolonged soaking and leaching treatments, repeated washing, and the application of traditional practices involving the use of clay or ash [29,32]. The process of cooking pasta resulted in a further reduction in tannin levels, attributable to two primary mechanisms: thermal degradation and the leaching of water-soluble tannins into the boiling water [36]. Tannins are classified as antinutrients due to their mineral-chelating capacity and their potential to reduce nutrient bioavailability. However, they are generally not considered a major dietary concern under normal consumption, except for populations vulnerable to nutritional deficiencies. A suggested safe daily intake threshold is 1.5–2 g [37]. The tannin content in cooked A50 and A100 was comparable to other acorn-enriched products, such as *focaccia* formulated with 30% AF [38] and biscuits with 41% AF [39]. From a consumer perspective, tannins are often considered more relevant for their potential impact on sensory properties, particularly bitterness and astringency. In this case, these sensory notes were only slightly perceived but did not compromise the palatability of the pasta.

### 2.3. Texture, Cooking Performance, and Color

Texture is a crucial physical attribute that has a significant impact on consumer acceptance of pasta. In the context of Italian culinary tradition, the ideal way to cook pasta is defined as “*al dente*”, a term that indicates a certain degree of resistance when chewing, resulting in pasta that is cooked but still firm [40]. This way of cooking is generally associated with reduced digestibility and a lower glycemic index, as the starch structure remains partially ungelatinized [41]. Among the three formulations, there were no differences in the optimal cooking time (Table 3).

The texture of *tagliatelle* was evaluated using a cutting test. This mimics the mechanical action of teeth during mastication, measuring the force required to cut cooked pasta (Table 3). The incorporation of AF resulted in a significantly higher cutting force, indicating a firmer texture. This effect could be related to differences in the amylose-to-amylopectin ratio and their associated physicochemical properties, as well as to the gelatinization behavior of acorn starch and starch–protein interactions [42]. In addition, the high fiber content in AF could be a factor in the increased firmness, which translates into a more rigid structural network [31].

The other added value of acorns, besides their fiber content, is mainly related to their polyphenol content. As discussed previously in the rheological analysis results, polyphenols may have interacted with starch molecules, acting as structural modifiers and influencing the texture of cooked pasta. In fact, starch–polyphenol complexes can form through hydrogen bonding, thereby altering the structural organization and functional properties of the starch matrix [43].

Sample A100 demonstrated the highest values for both WAI and SI. This phenomenon can be due to the high fiber content of AF. In fact, fiber can retain water and swell, thereby contributing significantly to the pasta’s overall water uptake during cooking. The observed cooking losses were significantly lower than the maximum 8–12% limit established for good-quality pasta [44,45]. Nevertheless, A100 showed a significantly higher cooking loss than A50. While this difference was statistically significant (*p* ≤ 0.05), its practical relevance for consumer acceptance is hypothesized to be limited. As both samples remained below the threshold at which cooking loss is generally associated with notable deterioration in pasta quality attributes such as firmness, stickiness, and cooking water turbidity, the results can be explained by the weakening of the dough structure resulting in more starch and soluble solids being released into the cooking water. A similar phenomenon has been observed in formulations with high fiber content or non-traditional ingredients, where the binding network is less efficient at retaining solids than in gluten-based formulations [44]. In accordance with these considerations, Konya et al. [15] have reported analogous behavior in gluten-free egg-based noodles, reaching a value of 9.28% in the formulation containing 40% AF.

**Figure 4:**
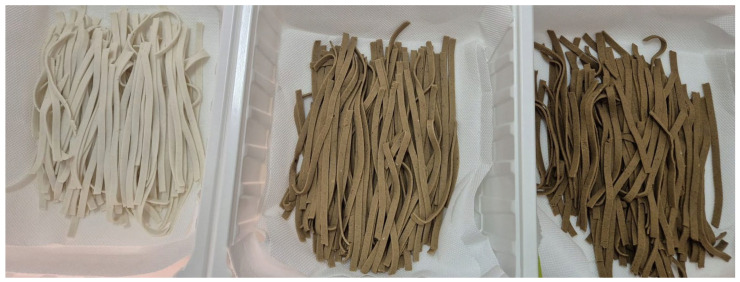
Experimental *tagliatelle* pasta samples. From left to right: CTRL = *tagliatelle* 100% rice flour; A50 = *tagliatelle* 50% rice flour–50% acorn flour; A100 = *tagliatelle* 100% acorn flour.

The inclusion of AF significantly changed the pasta color, shifting it away from the white of RF (Table 4 and Figure 4). This chromatic effect could be positively perceived by contemporary consumers, who have demonstrated an increasing preference for colored pasta varieties enriched with vegetable powders, cuttlefish ink, or other alternative flours [46,47]. This is particularly evident in gluten-free versions, which are available on the market in bright colors, such as green, orange, and blue, caused by the use, for example, of pea flour, red lentil flour and spirulina [48,49,50,51]. Incorporating AF significantly reduced lightness, giving the pasta a darker hue. AF increased the redness, yellowness and browning of the pasta. These color properties were due to the natural pigments present in AF in both the raw and cooked pasta samples, which also suggests good stability throughout the cooking process. The brown color of AF is typically caused by the activity of polyphenol oxidases. These enzymes oxidize phenolic compounds, producing dark pigments known as *o*-quinones. They can also participate in other reactions, complexing with other constituents and producing other brown polymers [52,53]. For both raw and cooked pasta, ΔE values were significantly higher than 3, a value generally considered to be easily appreciable by the human eye. Consequently, the color differences among the samples were sufficiently pronounced to be easily perceived by consumers under normal observation conditions [54].

### 2.4. Quantitative Descriptive Sensory Analysis

**Figure 5:**
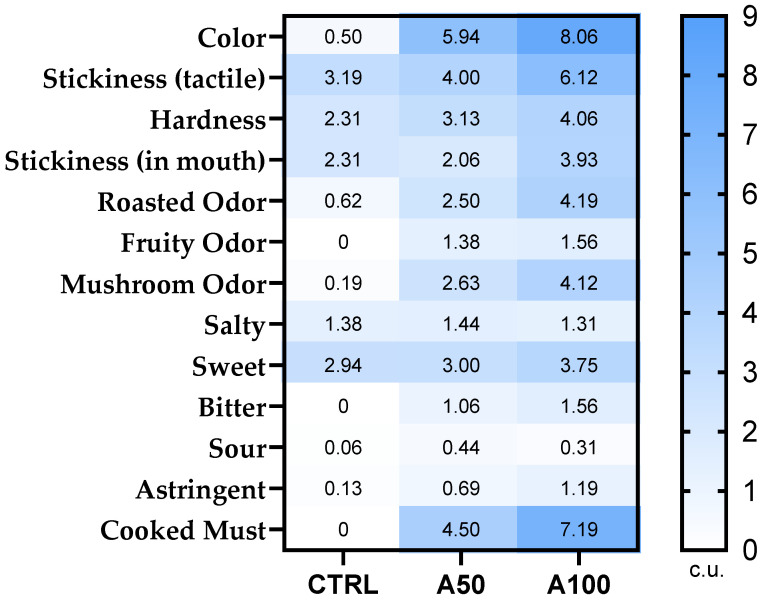
Heat map of the quantitative descriptive sensory analysis of *tagliatelle* pasta samples. CTRL = *tagliatelle* 100% rice flour; A50 = *tagliatelle* 50% rice flour–50% acorn flour; A100 = *tagliatelle* 100% acorn flour. c.u. = contractual units. Data are expressed as means of three replicates.

The sensory acceptability of gluten-free pasta has long been considered deficient in terms of both structure and taste, despite recent improvements [55,56,57]. Figure 5 shows the results of a sensory analysis considering the 13 sensory attributes evaluated on cooked *tagliatelle* made with hydrogel, RF, and/or AF.

Overall, incorporating AF modified the sensory parameters of the pasta compared to the formulation made with 100% RF. The sensory analysis was consistent with the instrumental evaluations for color and texture: the AF caused a brown-colored pasta and greater firmness.

The higher perceived adhesiveness, both on the fingers and in the mouth, can be attributed to the combined effect of fiber and starch gelatinization. The presence of fiber has been hypothesized to have reduced the hydrogel network’s capacity to retain starch granules and soluble solids during cooking, resulting in a greater amount of gelatinized starch on the product surface. These observations also reflect the higher cooking loss observed for samples with AF.

In terms of olfactory and flavor perception, the AF imparted notes reminiscent of fruit, mushrooms, cooked grape must and fig must. These are characteristic sensory notes of AF, also described as having a cashew and chocolate hint [58]. However, these sweet and earthy notes are unexpected in pasta, generally neutral in odor and flavor. Therefore, the selection of condiments should consider these notes, trying to adapt and have a balanced flavor [59]. In addition, the choice of sauce could be used to mask the slight bitterness typical of AF, caused by tannins [32]. Future studies will include a consumer test to validate product acceptability and to improve the robustness and reliability of the sensory findings.

## 3. Conclusions

The use of egg substitutes and gluten alternatives has received global attention, primarily due to vegan trends, animal welfare concerns, and the growing interest from individuals affected by allergies or intolerances. This study proposes a gluten-free and egg-free *tagliatelle* formulation, based on a carob–xanthan hydrogel combined with AF, using RF pasta as a control.

Rheological analyses showed that AF promoted a more pronounced elastic behavior, as indicated by the higher G′ modulus. Formulations containing AF exhibited increased lipid, ash, and fiber contents, with the 100% AF sample qualifying as a “source of fiber” according to EC Reg. 1924/2006 [30]. AF also enhanced the antioxidant profile of *tagliatelle*, particularly in terms of phenolic compounds and carotenoids.

The incorporation of AF influenced several characteristics, including color, texture and cooking properties. The sensory analysis confirmed the instrumental analyses, revealing higher odor and flavor complexity compared with the formulation prepared exclusively with RF.

The results of this study showed that the incorporation of hydrogel is a promising strategy for reproducing the structural roles of eggs and gluten in *tagliatelle* formulation. Moreover, AF has the potential to offer a novel alternative, thereby adding value to this natural and sustainable ingredient.

Future perspectives will investigate the nutritional implications of these formulations, including starch fractionation (rapidly digestible starch, slowly digestible starch and resistant starch), as well as their glycemic impact and related metabolic responses. Future research will also focus on sensory benchmarking against commercial *tagliatelle* and consumer acceptance tests to define appropriate sauce pairings that complement the unique flavor profile of acorn-enriched pasta. This will facilitate a more precise definition of the product’s sensory performance, consumer relevance, and overall commercial potential.

In conclusion, the synergy between carob–xanthan hydrogels and AF contributes to the development of a viscoelastic network with structural functionalities typically associated with gluten and eggs, while also transforming *tagliatelle* into a high-fiber, antioxidant-rich, clean-label functional food. By successfully integrating a hydrogel system with AF, this study provides a validated framework for developing gluten-free and vegan pasta with enhanced rheological complexity and nutritional value, laying the groundwork for future glycemic and shelf-life optimizations. From a practical perspective, these findings offer the food industry a scalable strategy for formulation optimization, demonstrating how hydrogels can effectively mimic the structural roles of gluten and eggs without compromising pasta quality. Furthermore, the utilization of AF paves the way for the valorization of this underutilized, sustainable resource. Given current global trends, this innovative formulation holds significant market potential, offering a nutritionally balanced alternative that aligns with modern consumer preferences.

## 4. Materials and Methods

### 4.1. Ingredients and Process

Rice flour (RF) (Lo Conte, Rome, Italy) (fat 0.5 g/100 g; carbohydrates 82 g/100 g; fiber 0.5 g/100 g; protein 7 g/100 g), xanthan gum (100% xanthan gum) (Food Bites, Rovello Porro, Italy) and carob seed flour (100% carob seed flour) (Rapunzelstraße, Legau, Germany) were purchased from local retailers. Commercial acorn flour (AF) (*Quercus rotundifolia*) was provided by Landratech (Manhente, Portugal) (fat 11.8 g/100 g; carbohydrates 75 g/100 g; fiber 3 g/100 g; protein 5.5 g/100 g).

For the hydrogel formulation, 100 mL of water was added to 2.5 g xanthan gum and 2.5 g carob seed flour and mixed with an immersion blender (Kenwood, London, UK) for 3 min. The gel was then used in the formulation of pasta, minimally modifying the ratio between gel and flour reported in Costantini et al. [17] (Table 5).

Dough was hand-mixed over a period of 20 min. Then, the dough was allowed to rest for 15 min at 20 °C, and hand-mixed for another 5 min. The pasta was extruded through a die to obtain its shape, namely *tagliatelle* (Torkio Ok Leonardo, Franceschi, Anzola dell’Emilia, Italy). The *tagliatelle* had a thickness of 2 mm, a width of 1 cm and a length of 10 cm. Three batches were prepared for each formulation.

### 4.2. Rheological Properties of Dough

The rheological properties of dough were evaluated with a rheometer (Haake Mars iQ Air, Thermo Fisher Scientific, Waltham, MA, USA) equipped with a parallel plate geometry (P35/Ti 02180932), as reported by Renoldi et al. [60] with minimal modifications. For the frequency sweep test a gap was set at 3 mm, the frequency in the ramp 0.1–10 Hz, at 20 °C at a strain value of 2.0%, within the linear viscoelastic region (LVR) of doughs. The storage modulus (G′) and loss modulus (G″) were recorded. The tan δ was calculated as the ratio between G″ and G′ at 1 Hz.

For the temperature sweep test the following conditions were considered: 2.0% strain, 1 Hz of frequency, heating ramp 25 to 90 °C, heating rate 3 °C/min, according to Peressini et al. [61], with minor modifications. The sample was covered during the analysis, using a sample hood to prevent water evaporation. The G′ modulus was recorded.

The rheological analyses were carried out in triplicate.

### 4.3. Proximate Composition and Water Activity

For moisture, a moisture analyzer at 105 °C (Radwag Wagi Elektroniczne, Radom, Poland) was used, in line with the AACC method 44–01.01 [62]. The water activity (a_w_) was measured using a hygrometer (Aqua Lab 100–240 V AC, Pullman, WA, USA).

The protein and ash contents were determined as described by the AACC methods 46–11.02 and 08–01.01 [62], respectively. The lipids were extracted and quantified using diethyl ether as the extraction solvent, with a semi-automatic extraction system (Velp Scientifica srl, Usmate, Italy) according to the AOAC method 922.06 [63]. The total dietary fiber was determined according to the AOAC method 991.43 [63]. The carbohydrates were calculated by subtracting the moisture, protein, lipid, and ash contents from 100. The results were expressed as g/100 g. The macronutrients were multiplied for the conversion factors (4 kcal/g for protein and carbohydrates, 9 kcal/g for lipids, and 2 kcal/g for fiber) and summed to obtain the energy value. Three replicates were carried out for each determination.

### 4.4. Bioactive Compounds and Antioxidant Activity

Extraction and quantification of total phenolic compounds (TPC), ABTS (2,2′-azino-bis-3-ethyl benzthiazoline-6-sulphonic acid) and DPPH (2,2-diphenyl-1-picrylhydrazyl) in vitro antioxidant activity assays were performed in triplicate, according to Vurro et al. [54].

The total carotenoid pigments were extracted, in triplicate, using water-saturated *n*-butyl alcohol. Then the absorbance of the supernatants was read at 435.8 nm by a Cary 60 UV–Vis spectrophotometer (Agilent Technologies, Santa Clara, CA, USA), according to the AACC method 14–50.01 [62].

The tannins were quantified by minimally modifying the method described by Monteiro [64]. The extraction of tannins was performed under the same conditions applied for the TPC and antioxidant activity. Briefly, for the control, 1 mL of sample extract was mixed with 2 mL of saturated solution of (NH_4_)_2_SO_4_ and 7 mL of deionized water (ELGA Purelab, High Wycombe, UK). In parallel, 1 mL of extract was mixed with 3 mL of 0.04% methylcellulose solution, 2 mL of saturated (NH_4_)_2_SO_4_, and 4 mL of deionized water. After 10 min at 20 °C, in the dark, the tubes were centrifuged (Thermo Fisher Scientific, Osterode am Harz, Germany) at 10,000× *g* at 4 °C. The absorbance of both supernatants was recorded at 280 nm using quartz cuvettes. Tannin content was calculated considering the difference in the absorbances and expressed as mg epicatechin equivalents (EE)/g d.m. The analysis was carried out in triplicate.

### 4.5. Texture, Cooking Performance, and Color Analysis

First, the optimal cooking time (OCT) was established for all samples according to the AACC method 66 [62]. Briefly, 20 g of *tagliatelle* were cooked in 200 mL of boiling unsalted distilled water. At 30 s intervals, samples were removed and cross-sectionally cut to visually observe the disappearance of the central opaque core.

The textural properties of cooked pasta at OCT, rinsed with distilled water and drained, were evaluated using a cutting test, with a texture analyzer equipped with a 1 kN load cell (Z1.0 TN, Zwick Roell, Ulm, Germany) according to Costantini et al. [17]. Four replicates were carried out for each batch.

The cooking performances, namely the swelling index (SI) and water absorption index (WAI), were determined in triplicate, according to Wang et al. [65]. Cooking loss (CL) was determined as reported by AACC method 66 [62], expressing the results as a percentage.

The color was measured using a colorimeter (CM-600d, Konica Minolta, Tokyo, Japan) equipped with SpectraMagic NX software (Konica Minolta, Tokyo, Japan). Before the measurement process, the instrument was calibrated using a standard white calibration tile, in accordance with the CIE (Commission Internationale de l’Éclairage) Illuminant C condition. The lightness (L*, from black to white), redness index (a*, from green to red), and yellowness index (b*, from blue to yellow) were determined in the CIE color space. The brown index was calculated as (100 L*). The color difference (∆E) was calculated according to Vurro et al. [54], using the same color evaluation scale. Six replicates were carried out for each batch.

### 4.6. Quantitative Descriptive Analysis

The sensory analysis of *tagliatelle* was performed considering the quantitative descriptive analysis (QDA), in accordance with the International Standardization Organization (ISO) standard 13299 [66] by a trained panel of eight people (four men and four women, ages 20 to 60). The members of the panel gave written approval in accordance with the ethical norms of the Laboratory of Food Science and Technology, Department of Soil, Plant and Food Science, University of Bari (Bari, Italy). They had no dietary intolerances or allergies. The panel was trained in accordance with ISO standards [66,67] for descriptor familiarization, evaluation rigor, and performance monitoring. A pre-test session was carried out according to ISO Standard 11132 [67]. A scale of intensity from 0 to 9 contractual units (c.u.) was used. Table 6 reports the descriptors considered. Fifteen-gram samples of cooked *tagliatelle* (OCT) were served in white dishes to each panelist for sensory evaluation. The samples were coded and presented in random order. The test was performed in the sensory analysis laboratory belonging to the Food Science and Technology Laboratory, Department of Soil, Plant and Food Science, University of Bari (Bari, Italy) [68]. Three replicates were carried out.

### 4.7. Statistical Analysis

The results were expressed as mean standard deviation (SD). Statistical analysis was performed using Minitab Statistical Software 21 (Minitab Inc., State College, PA, USA). The significant differences (*p* ≤ 0.05) were assessed by one-way parametric analysis of variance (ANOVA), followed by the Tukey HSD test. Graphs were created using GraphPad Prism 10 (GraphPad Software, San Diego, CA, USA).

## Figures and Tables

**Table 1:** Nutritional composition and water activity (a_w_) of *tagliatelle* pasta samples.

g/100 g	CTRL	A50	A100
Moisture	35.03 ± 0.53 a	34.55 ± 0.99 ab	33.05 ± 0.12 b
Lipid	0.76 ± 0.06 c	4.56 ± 0.17 b	8.11 ± 0.13 a
Protein	6.03 ± 0.09 a	4.55 ± 0.53 b	3.53 ± 0.08 c
Carbohydrates	57.85 ± 0.40 a	55.54 ± 0.53 ab	54.20 ± 0.07 b
Fiber	2.29 ± 0.08 c	2.69 ± 0.17 b	4.37 ± 0.20 a
Ashes	0.34 ± 0.03 c	0.79 ± 0.01 b	1.12 ± 0.14 a
Energetic value (kcal/100 g)	257.70 ± 10.64 c	276.10 ± 4.49 b	295.20 ± 0.14 a
a_w_	0.97 ± 0.00 a	0.96 ± 0.01 a	0.96 ± 0.03 a

Data are presented as means ± SD of three replicates. Different letters within the same row indicate significant differences (*p* ≤ 0.05). CTRL = *tagliatelle* 100% rice flour; A50 = *tagliatelle* 50% rice flour–50% acorn flour; A100 = *tagliatelle* 100% acorn flour.

**Table 2:** Tannin content of raw and cooked *tagliatelle* pasta samples.

Sample	Raw mg EE/g d.m.	Cooked mg EE/g d.m.
CTRL	0.66 ± 0.01 c	n.d.
A50	10.33 ± 0.41 b	6.54 ± 0.12 b
A100	17.99 ± 0.51 a	8.02 ± 0.12 a

Data are presented as means ± SD of three replicates. Different letters within the same column indicate significant differences at *p* ≤ 0.05. CTRL = *tagliatelle* 100% rice flour; A50 = *tagliatelle* 50% rice flour–50% acorn flour; A100 = *tagliatelle* 100% acorn flour. d.m. = dry matter; EE = epicatechin equivalent; n.d. = not detected.

**Table 3:** Textural features and cooking performance of *tagliatelle*.

Sample	OTC (Min)	Hardness (N)	WAI (g/100 g)	SI (g Water/g Pasta)	Cooking Loss (g/100 g)
CTRL	2.0	25.32 ± 0.70 c	37.52 ± 2.08 b	1.14 ± 0.03 b	2.33 ± 0.24 c
A50	2.0	32.51 ± 0.94 b	41.95 ± 2.12 b	1.32 ± 0.07 b	3.71 ± 0.32 b
A100	2.0	39.12 ± 0.68 a	54.00 ± 2.97 a	1.60 ± 0.12 a	5.56 ± 0.52 a

Data are reported as means ± SD of four replicates for texture analysis and three replicates for cooking performance. Different letters within the same column indicate significant differences (*p* ≤ 0.05). CTRL = *tagliatelle* 100% rice flour; A50 = *tagliatelle* 50% rice flour–50% acorn flour; A100 = *tagliatelle* 100% acorn flour; OCT = Optimal Cooking Time; WAI = Water Absorption Index; SI = Swelling Index.

**Table 4:** Color of raw and cooked *tagliatelle* pasta samples.

	CTRL	A50	A100
Raw			
L*	89.25 ± 0.50 a	65.6 ± 3.14 b	54.00 ± 2.82 c
a*	−0.32 ± 0.07 c	6.19 ± 0.07 b	8.25 ± 0.06 a
b*	8.22 ± 0.10 b	24.19 ± 0.88 a	24.95 ± 0.85 a
Brown index	10.75 ± 0.50 c	34.40 ± 3.14 b	46.00 ± 2.82 a
ΔE	-	29.9	39.9
Cooked			
L*	72.85 ± 2.30 a	51.83 ± 1.45 b	47.23 ± 0.72 c
a*	−0.92 ± 0.06 b	6.78 ± 0.24 a	6.99 ± 0.10 a
b*	6.62 ± 0.65 b	18.21 ± 0.48 a	19.01 ± 0.50 a
Brown index	27.15 ± 2.30 c	48.18 ± 1.45 b	52.77 ± 0.72 a
ΔE	-	25.2	29.5

Data are expressed as means ± SD of six replicates. Different letters within the same row indicate significant differences (*p* ≤ 0.05). CTRL = *tagliatelle* 100% rice flour; A50 = *tagliatelle* 50% rice flour–50% acorn flour; A100 = *tagliatelle* 100% acorn flour.

**Table 5:** Formulation of experimental *tagliatelle* pasta samples.

Ingredient (g)	CTRL	A50	A100
Rice flour	100	50	0
Acorn flour	0	50	100
Gel *	61.5	61.5	61.5

* 2.5 g xanthan gum + 2.5 g carob seed flour + 100 g of tap water at 20 °C.

**Table 6:** Sensory descriptors of quantitative descriptive analysis.

Descriptor	Definition	Min. (0)	Max. (9)
Color	Color of pasta	White	Dark brown
Stickiness (tactile)	Stickiness when touching the product between the fingers	Absent	Very intense
Hardness	Tightness in cooking	Soft	Hard
Stickiness (in mouth)	Stickiness of the product in the mouth, on the palate	Absent	Very intense
Roasted odor	Typical odor associated with baked bread	Absent	Very intense
Fruity odor	Typical odor associated with ripe fruit	Absent	Very intense
Mushroom odor	Typical odor associated with mushroom	Absent	Very intense
Salty	Typical flavor related to sodium chloride	Absent	Very intense
Sweet	Typical flavor related to sucrose	Absent	Very intense
Bitter	Typical flavor related to caffeine	Absent	Very intense
Sour	Typical flavor related to citric acid	Absent	Very intense
Astringent	Typically related to tannins, unripe fruit	Absent	Very intense
Cooked must	Typical flavor related to cooked must, obtained from grapes and figs	Absent	Very intense

Scale: 0–9 contractual units (c.u.). 0 = minimum perceived; 9 = maximum perceived.

## Data Availability

Data are contained within the article.

## Abbreviations

RF	Rice Flour
AF	Acorn Flour
TPC	Total Phenolic Compounds
GAE	Gallic Acid Equivalent
TE	Trolox Equivalent
EE	Epicatechin Equivalent
WAI	Water Absorption Index
SW	Swelling Index
OCT	Optimal Cooking Time

## References

[1] Nazari V., Pasqualone A., Pieroni A., Todisco V., Belardinelli S., Pievani T. (2024). Evolution of the Italian *pasta ripiena*: The first steps toward a scientific classification. Discov. Food.

[2] Helcke K. (2016). Pasta and Noodles: A Global History.

[3] Roshini J., Pandey A.K., Vashishth R. (2025). History and Origin of Pasta. Advances in Pasta Technology.

[4] (2001). Decreto del Presidente della Repubblica 9 Febbraio 2001, n.187. Regolamento per la revisione della normativa sulla produzione e commercializzazione di sfarinati e paste alimentari, a norma dell’articolo 50 della legge 22 febbraio 1994, n. 146. Official Journal of the Italian Republic.

[5] Hassoun A., Boukid F., Pasqualone A., Bryant C.J., García G.G., Parra-López C., Jagtap S., Trollman H., Cropotova J., Barba F.J. (2022). Emerging trends in the agri-food sector: Digitalisation and shift to plant-based diets. Curr. Res. Food Sci..

[6] Pilař L., Stanislavská L.K., Kvasnička R., Hartman R., Tichá I. (2021). Healthy food on Instagram social network: Vegan, homemade and clean eating. Nutrients.

[7] Saini P., Kaur H., Tyagi V., Saini P., Ahmed N., Dhaliwal H.S., Sheikh I. (2023). Nutritional value and end-use quality of durum wheat. Cereal Res. Commun..

[8] Zhang H., Zhang F., Yuan R., Chen Y. (2020). Applications of natural polymer-based hydrogels in the food industry. Hydrogels Based on Natural Polymers.

[9] Gadoum A., Difallah A., Adda A., Merah O. (2026). Carob Tree: A Review of Traditional Uses, Medicinal Properties, and Future Perspectives in Sustainable Forestry. Life.

[10] Berninger T., Dietz N., González López Ó. (2021). Water-soluble polymers in agriculture: Xanthan gum as eco-friendly alternative to synthetics. Microb. Biotechnol..

[11] Zheng Z., Sun Z., Li M., Yang J., Yang Y., Liang H., Yang S. (2024). An update review on biopolymer Xanthan gum: Properties, modifications, nanoagrochemicals, and its versatile applications in sustainable agriculture. Int. J. Biol. Macromol..

[12] Manzoor A., Dar A.H., Pandey V.K., Shams R., Khan S., Panesar P.S., Khan S.A. (2022). Recent insights into polysaccharide-based hydrogels and their potential applications in food sector: A review. Int. J. Biol. Macromol..

[13] Stankov Jovanović V., Djurić V., Mitić V., Barjaktarević A., Cupara S., Ilić M., Nikolić J. (2025). Oak Acorns as Functional Foods: Antioxidant Potential and Safety Assessment. Foods.

[14] Zhang Q., Yu J., Li K., Bai J., Zhang X., Lu Y., Sun X., Li W. (2021). The rheological performance and structure of wheat/acorn composite dough and the quality and in vitro digestibility of its noodles. Foods.

[15] Konya S., Aktaş K. (2024). The quality assessment of starch based noodles enriched with acorn flour, cooking characteristics, physical, chemical and sensorial properties. Braz. Arch. Biol. Technol..

[16] Kasprzak-Drozd K., Mołdoch J., Gancarz M., Wójtowicz A., Kowalska I., Oniszczuk T., Oniszczuk A. (2024). In vitro digestion of polyphenolic compounds and the antioxidant activity of acorn flour and pasta enriched with acorn flour. Int. J. Mol. Sci..

[17] Costantini M., Summo C., Faccia M., Caponio F., Pasqualone A. (2021). *Kabuli* and *Apulian black* chickpea milling by-products as innovative ingredients to provide high levels of dietary fibre and bioactive compounds in gluten-free fresh pasta. Molecules.

[18] Larrosa V., Lorenzo G., Zaritzky N., Califano A. (2013). Optimization of rheological properties of gluten-free pasta dough using mixture design. J. Cereal Sci..

[19] Szabłowska E., Tańska M. (2021). Acorn flour properties depending on the production method and laboratory baking test results: A review. Compr. Rev. Food Sci. Food Saf..

[20] Yazar G., Demirkesen I. (2023). Linear and non-linear rheological properties of gluten-free dough systems probed by fundamental methods. Food Eng. Rev..

[21] Mostafa S., Ata S.M., Hussein A.M., Zaky A.A. (2025). Development and quality evaluation of high-protein gluten-free pasta formulations. Sci. Rep..

[22] Beltrão Martins R., Gouvinhas I., Nunes M.C., Alcides Peres J., Raymundo A., Barros A.I. (2020). Acorn flour as a source of bioactive compounds in gluten-free bread. Molecules.

[23] Guan Y., Wang Y., Yang X., Li L., Shi F., Li M., Li Y. (2025). Effects of tannic acid on physicochemical properties of gluten-free flour and the underlying mechanisms. Food Hydrocoll..

[24] Yoshimura M., Sugahara Y., Nagase K., Kobayashi M., Kamatari Y.O., Yamauchi K. (2025). Protein-tannins binding mode in hydrolysable tannins-induced protein aggregation. Food Chem..

[25] Mamet T., Yao F., Li K.K., Li C.M. (2017). Persimmon tannins enhance the gel properties of high and low methoxyl pectin. LWT.

[26] Correia P.R., Beirão-da-Costa M.L. (2011). Effect of drying temperatures on starch-related functional and thermal properties of acorn flours. J. Food Sci..

[27] Singh V., Okadome H., Toyoshima H., Isobe S., Ohtsubo K.I. (2000). Thermal and physicochemical properties of rice grain, flour and starch. J. Agric. Food Chem..

[28] Jiang H., Mcclements D.J., Dai L., Qin Y., Ji N., Xiong L., Qiu C., Sun Q. (2024). Effects of moisture content and retrogradation on structure and properties of indica rice flour and starch gels. Food Hydrocoll..

[29] Inácio L.G., Bernardino R., Bernardino S., Afonso C. (2024). Acorns: From an Ancient food to a modern sustainable resource. Sustainability.

[30] European Parliament and the Council (2006). Regulation (EC) No 1924/2006 of the European Parliament and of the Council of 20 December 2006 on nutrition and health claims made on foods. Off. J. Eur. Union.

[31] Szabłowska E., Tańska M. (2025). Effects of acorn flour addition on baking characteristics of wheat flour. Foods.

[32] Szabłowska E., Tańska M. (2024). Acorns as a Source of Valuable Compounds for Food and Medical Applications: A Review of *Quercus* Species Diversity and Laboratory Studies. Appl. Sci..

[33] Dello Russo M., Spagnuolo C., Moccia S., Angelino D., Pellegrini N., Martini D., Italian Society of Human Nutrition (SINU) Young Working Group (2021). Nutritional quality of pasta sold on the Italian market: The food labelling of Italian products (FLIP) study. Nutrients.

[34] Gezici S., Sekeroglu N. (2019). Neuroprotective potential and phytochemical composition of acorn fruits. Ind. Crops Prod..

[35] Shruthi P., Sajayan A., Thakur K., Kaur J., Sharma S., Sharma R., Gupta A., Bobade H. (2025). Antioxidant Rich Pasta. Advances in Pasta Technology.

[36] Dordevic D., Zemancova J., Dordevic S., Kulawik P., Kushkevych I. (2025). *Quercus* acorns as a component of human dietary patterns. Open Agric..

[37] Zayed A., Abdelkareem S., Talaat N., Dayem D.A., Farag M.A. (2025). Tannin in foods: Classification, dietary sources, and processing strategies to minimize anti-nutrient effects. Food Bioprocess Technol..

[38] Vurro F., Haddada A., Ailoaiei A.M., De Angelis D., Hedia H., Klibi N., Caponio G.R., Squeo G., Stambouli-Essassi S., Pasqualone A. (2026). Formulation of *focaccia* with acorn flour from Tunisian *Quercus canariensis* Willd: A strategy for lowering the glycemic index while enhancing nutritional value. Future Foods.

[39] Lukinac J., Medaković D., Koceva Komlenić D., Šušak A., Jukić M. (2026). Application of *Quercus pubescens* Acorn flour and xanthan gum in gluten-free cookies: RSM optimization and quality evaluation. Foods.

[40] Vilgis T.A., Toultchinski P. (2024). How to cook pasta? Physicists view on suggestions for energy saving methods. Phys. Fluids.

[41] Dodi R., Di Pede G., Scarpa C., Deon V., Dall’Asta M., Scazzina F. (2023). Effect of the Pasta Making Process on Slowly Digestible Starch Content. Foods.

[42] Wang J., Li Y., Guo X., Zhu K., Wu Z. (2024). A Review of the Impact of Starch on the Quality of Wheat-Based Noodles and Pasta: From the View of Starch Structural and Functional Properties and Interaction with Gluten. Foods.

[43] Wu Y., Liu Y., Jia Y., Zhang H., Ren F. (2024). Formation and Application of Starch–Polyphenol Complexes: Influencing Factors and Rapid Screening Based on Chemometrics. Foods.

[44] Romano A., Ferranti P., Gallo V., Masi P. (2021). New ingredients and alternatives to durum wheat semolina for a high quality dried pasta. Curr. Opin. Food Sci..

[45] Giuberti G., Gallo A., Cerioli C., Fortunati P., Masoero F. (2015). Cooking quality and starch digestibility of gluten free pasta using new bean flour. Food Chem..

[46] Sobota A., Wirkijowska A., Zarzycki P. (2020). Application of vegetable concentrates and powders in coloured pasta production. Int. J. Food Sci. Technol..

[47] Teterycz D., Sobota A., Zarzycki P., Latoch A. (2020). Legume flour as a natural colouring component in pasta production. J. Food Sci. Technol..

[48] Koli D.K., Rudra S.G., Bhowmik A., Pabbi S. (2020). Nutritional, functional, textural and sensory evaluation of Spirulina enriched green pasta: A potential dietary and health supplement. Foods.

[49] Raczyk M., Polanowska K., Kruszewski B., Grygier A., Michałowska D. (2022). Effect of spirulina (*Arthrospira platensis*) supplementation on physical and chemical properties of semolina (*Triticum durum*) based fresh pasta. Molecules.

[50] Bresciani A., Erba D., Casiraghi M.C., Iametti S., Marti A., Barbiroli A. (2022). Pasta from red lentils (*Lens culinaris*): The effect of pasta-making process on starch and protein features, and cooking behavior. Foods.

[51] Sudha M.L., Leelavathi K. (2012). Effect of blends of dehydrated green pea flour and amaranth seed flour on the rheological, microstructure and pasta making quality. J. Food Sci. Technol..

[52] Tilley A., McHenry M.P., McHenry J.A., Solah V., Bayliss K. (2023). Enzymatic browning: The role of substrates in polyphenol oxidase mediated browning. Curr. Res. Food Sci..

[53] Zhang S. (2023). Recent Advances of Polyphenol Oxidases in Plants. Molecules.

[54] Vurro F., Anelli P., Zocchi D.M., De Bellis P., Pieroni A., Pasqualone A. (2025). Bessarabian wild hop sourdough: Microbial characterization and effect on the physicochemical properties and flavor of the bread. Int. J. Gastron. Food Sci..

[55] Marti A., Pagani M.A. (2013). What can play the role of gluten in gluten free pasta?. Trends Food Sci. Technol..

[56] Xhakollari V., Daniele G.M., Cianciabella M., Medoro C., Paradiso V., Canavari M. (2025). Exploring the sensory characteristics and understanding consumer acceptance of gluten-free pasta. Eur. Food Res. Technol..

[57] Capriles V.D., de Aguiar E.V., Dos Santos F.G., Fernández M.E.A., de Melo B.G., Tagliapietra B.L., Conti A.C. (2023). Current status and future prospects of sensory and consumer research approaches to gluten-free bakery and pasta products. Food Res. Int..

[58] Bainbridge D.A. (2001). Acorns as Food: History, Use, Recipes, and Bibliography.

[59] Ribes S., Gómez-Llorente H., Córdoba L., Fernández-Segovia I., Albors A., Barat J.M., Pérez-Esteve É. (2025). Carob (*Ceratonia siliqua* L.) flour as a functional ingredient in fresh wheat pasta: Effect on its technological and sensory properties, oral processing and in vitro health benefits. Food Res. Int..

[60] Renoldi N., Brennan C.S., Lagazio C., Peressini D. (2021). Evaluation of technological properties, microstructure and predictive glycaemic response of durum wheat pasta enriched with psyllium seed husk. LWT.

[61] Peressini D., Cavarape A., Brennan M.A., Gao J., Brennan C.S. (2020). Viscoelastic properties of durum wheat doughs enriched with soluble dietary fibres in relation to pasta-making performance and glycaemic response of spaghetti. Food Hydrocoll..

[62] American Association of Cereal Chemists (AACC) International (2010). Approved Methods of Analysis.

[63] Association of Official Agricultural Chemists (AOAC) International (2006). Official Methods of Analysis of AOAC International.

[64] Monteiro V., Soares C., Grosso C., Delerue-Matos C., Ramalhosa M.J. (2023). From forest to table: Optimizing the nutritional value of acorns through effective tannin extraction. Biol. Life Sci. Forum.

[65] Wang J., Brennan M.A., Brennan C.S., Serventi L. (2021). Effect of vegetable juice. puree. and pomace on chemical and technological quality of fresh pasta. Foods.

[66] (2016). Sensory Analysis—Methodology—General Guidance for Establishing a Sensory Profile.

[67] (2021). Sensory Analysis—Methodology—Guidelines for the Measurement of the Performance of a Quantitative Descriptive Sensory Panel.

[68] (2007). Sensory Analysis—General Guidance for the Design of Test Rooms.

